# Functional characterization of a novel class A carbapenemase CAE-1 in carbapenem-resistant *Pseudomonas aeruginosa* clinical isolates

**DOI:** 10.1128/aac.01362-25

**Published:** 2026-02-23

**Authors:** Jinhong Chen, Zhewei Sun, Jiachun Su, Pei Li, Xiaogang Xu, Minggui Wang

**Affiliations:** 1 Shanghai Institute of Infectious Disease and Biosecurity, Fudan University 12478, Shanghai, China; 2 Institute of Antibiotics, Huashan Hospital, Fudan University 159397, Shanghai, China; 3 Key Laboratory of Clinical Pharmacology of Antibiotics, National Health Commission of the People's Republic of China 198171, Shanghai, China; 4 Department of Laboratory Medicine, Huashan Hospital, Fudan University 33132 , Shanghai, China; Entasis, Big Bay, Michigan, USA

**Keywords:** carbapenem-resistant *Pseudomonas aeruginosa*, novel carbapenemase, enzyme kinetics, horizontal transfer, whole-genome sequencing

## Abstract

Two carbapenem-resistant *Pseudomonas aeruginosa* (CRPA) isolates were collected, which lacked known carbapenemases but produced a β-lactamase CAE-1. CAE-1 was recently found in *Comamonas aquatica* and conferred resistance to penicillins and cephalosporins. In this study, we aim to know whether CAE-1 was responsible for the carbapenem resistance in the CRPA isolates and its enzyme hydrolyzing characteristics. Both CRPA clinical isolates exhibited minimal inhibitory concentrations (MICs) of 8 µg/mL for imipenem and meropenem, with a positive result in the modified Hodge test for meropenem. The *bla*_CAE-1_ and *bla*_KPC-2_ were cloned and expressed in *P. aeruginosa* PAO1 and *Escherichia coli* DH5α, respectively. The *bla*_CAE-1_-carrying PAO1 transformant also tested positive using the modified carbapenem inactivation method and was resistant to piperacillin-tazobactam, ticarcillin-clavulanate, and cefoperazone-sulbactam, but susceptible to ceftazidime-avibactam. Expression of *bla*_CAE-1_ in *P. aeruginosa* PAO1 elicited a 64- to 128-fold increase in MICs for piperacillin, ceftazidime, cefepime, and aztreonam, and an eightfold increase for meropenem, exhibiting a broader resistance profile than in *E. coli* DH5α. Steady-state kinetic assays showed that CAE-1 had catalytic efficiency against all β-lactams tested, with comparatively lower efficiency against three carbapenems relative to KPC-2, while demonstrating approximately equivalent efficiency for the other β-lactam antibiotics tested. Whole-genome sequencing revealed that *bla*_CAE-1_ was a class A β-lactamase and encoded on an integrative and conjugative element, which might facilitate its horizontal transfer. The class A β-lactamase CAE-1 is a carbapenemase posing a high risk for horizontal dissemination. Enhanced surveillance for *bla*_CAE-1_-harboring isolates is needed.

## INTRODUCTION

*Pseudomonas aeruginosa* causes common, life-threatening infections and exhibits rising resistance to carbapenem antibiotics (1–3). Carbapenem-resistant *P. aeruginosa* (CRPA) has become a global threat due to a high mortality rate of 20%–30% (4). An important mechanism for the development of CRPA is the presence of carbapenemases, which can hydrolyze many β-lactams (5). The types of carbapenemases present in *P. aeruginosa* vary by region and include class A serine-β-lactamases (KPC and GES), class B metallo-β-lactamases (IMP, NDM, and AFM), and class D serine-β-lactamases (OXA-48) (6). Historically, carbapenemase detection rates in CRPA ranged from 10% to 20%, predominantly involving class B metallo-β-lactamases (7, 8). Our research group previously determined the enzymatic hydrolysis activity of the metalloenzyme AFM-1 from a clinical CRPA isolate (9). In recent years, the carbapenemase detection rate in CRPA has risen to 22% (7). Notably, KPC-2, a class A serine β-lactamase commonly found in carbapenem-resistant *Klebsiella pneumoniae*, has been transferred via plasmids into CRPA strains and now represents the most prevalent carbapenemase, with a detection rate of 49% (7, 10). The porin OprD is the principal channel protein through which imipenem enters *P. aeruginosa*; therefore, its functional inactivation can mediate imipenem resistance (11).

Recently, a novel class A serine β-lactamase, designated CAE-1, was identified on a plasmid from a strain of *Comamonas aquatica* isolated from sewage. Cloning assay in *Escherichia coli* demonstrated that CAE-1 confers resistance to ampicillin, piperacillin, cefazolin, cefuroxime, and ceftriaxone, but not to carbapenems. Searching the *bla*_CAE-1_ sequence in the NCBI Nucleotide database revealed that *bla*_CAE-1_ could be detected in *P. aeruginosa, C. aquatica, Comamonas thiooxydans,* and *Brevundimonas* sp. The latter three species were recognized as opportunistic human pathogens and predominantly found in environmental sources, such as sewage (12–14).

In this study, we identified two clinical CRPA isolates, PA56381 and PA56391, from two hospitalized patients. Whole-genome sequencing (WGS) did not detect any known carbapenemase genes; however, both isolates were found to carry *bla*_CAE-1_. Both isolates tested positive in the modified Hodge test. Cloning of *bla*_CAE-1_ from the clinical CRPA isolate into *P. aeruginosa* PAO1 resulted in resistance to piperacillin, ceftazidime, cefepime, and aztreonam, with minimal inhibitory concentrations (MICs) ranging from 64 to 256 µg/mL, and reduced susceptibility (intermediate) to meropenem (MIC = 4 µg/mL). Kinetic studies showed CAE-1 had catalytic efficiency against all β-lactams tested, including carbapenems. The above results suggest that CAE-1 is a carbapenemase and it contributed to reduced carbapenem susceptibility in combination with porin loss in *P. aeruginosa* clinical isolates. Comparative genomic analysis suggested that *bla*_CAE-1_ could be horizontally transferred through integrative and conjugative elements (ICEs) or plasmids and is already disseminated among clinical *P. aeruginosa* isolates.

## MATERIALS AND METHODS

### Bacterial strains and antimicrobial susceptibility testing

Clinical isolates of CRPA, PA56381 and PA56391, were recovered from a tertiary teaching hospital in Shanghai in 2019. Species identification was performed using MALDI-TOF MS. The MICs of 15 antibiotics were determined by the broth microdilution method, and the results were interpreted following the CLSI guidelines (15). *P. aeruginosa* ATCC 27853 was used as the quality control strain. Phenotypic detection of carbapenemase activity was conducted using a modified Hodge test (16). To verify carbapenemase production, the modified carbapenem inactivation method (mCIM) was performed according to CLSI guidelines (15). The test was conducted on the *P. aeruginosa* PAO1 transformant harboring *bla*_CAE-1_, utilizing the empty vector transformant *P. aeruginosa* PAO1 (pUCP24) and *K. pneumoniae* ATCC BAA-1705 as negative and positive controls, respectively. Porin phenotype experiments of two clinical strains were analyzed against the complete OprD porin of PAO1 reference, which served as the wild-type control (17).

### Cloning of *bla*_CAE-1_

In order to express *bla*_CAE-1_ in *P. aeruginosa* PAO1 and *E. coli* DH5α, the full-length 909 bp *bla*_CAE-1_ and its upstream 142 bp promoter region were co-amplified from the chromosome by PCR (primers shown in Table S1). The PCR products were digested with HindIII and BamHI and then ligated into the *E. coli–P. aeruginosa* shuttle vector pUCP24. The resulting recombinant plasmid, pUCP24-*bla*_CAE-1_, was initially transformed into *E. coli* DH5α. Transformants were selected on Luria-Bertani agar plates containing 50 µg/mL gentamicin and confirmed by PCR and Sanger sequencing. Subsequently, pUCP24-*bla*_CAE-1_ was electroporated into *P. aeruginosa* PAO1 and selected on LBA plates with 50 µg/mL gentamicin. To compare the resistance levels of *bla*_CAE-1_ to carbapenems and other antibiotics, *bla*_KPC-2_, a common class A carbapenemase in *P. aeruginosa* and Enterobacterales, was selected as a reference (18). The *bla*_KPC-2_ with the 142 bp upstream promoter was cloned into plasmid pUCP24, and the resulting recombinant plasmid pUCP24-*bla*_KPC-2_ was transferred into *P. aeruginosa* PAO1 and *E. coli* DH5α (Table S1). These transformants were then used for antimicrobial susceptibility testing. *E. coli* ATCC 25922 and *P. aeruginosa* ATCC 27853 were used as quality control strains. To compare the expression levels of *bla*_CAE-1_, total RNA was extracted from logarithmic-phase cultures of *E. coli* DH5α and *P. aeruginosa* PAO1 transformants. Quantitative real-time PCR (RT-qPCR) was performed to determine the transcriptional levels. The housekeeping genes *mdh* and *rpsL* were used as internal controls for *E. coli* and *P. aeruginosa*, respectively. Relative expression levels were analyzed to assess transcriptional differences between the species.

### Kinetic measurements of CAE-1

The *bla*_CAE-1_ and *bla*_KPC-2_ genes were cloned into the plasmid pET28a (+) vector, and the resulting plasmids were transformed into *E. coli* BL21 (DE3) (Table S1). CAE-1 and KPC-2 proteins were purified using His60 Ni Superflow Resin (Takara, Japan), with the protein purity (>90% homogeneity) confirmed by SDS-PAGE. Steady-state kinetic parameters *k*cat and *K*m were measured at 25°C with a UV-2700 spectrophotometer (Shimadzu, Kyoto, Japan) in 0.5 mL of 50 mM sodium phosphate buffer containing 50 μM ZnSO_4_ at pH 7.4 (9). The data are presented as the mean ± standard deviation based on three independent measurements.

### Bioinformatic analysis

Short-read sequencing of isolates PA56381 and PA56391 was conducted at the Beijing Genomics Institute (Illumina HiSeq X platform). Additionally, isolate PA56381 was subject to long-read sequencing at Shanghai Personal Biotechnology Co. Ltd. (Oxford Nanopore, Oxford, U.K.). Genomes were assembled using Unicycler v0.5.0 (19). We determined the cgMLST of 13 CAE-1 producers (11 from public database) with the homemade pipeline pyMLST (https://github.com/bvalot/pyMLST) using a core genome of 3,831 previously defined genes (20). Core genome alignment of 19 ST360 isolates was generated using snippy v4.6.0 (https://github.com/tseemann/snippy) and Gubbins v3.3.0 (https://github.com/nickjcroucher/gubbins), and a maximum likelihood phylogenetic tree was constructed using RAxML v8.2.4 (21).

## RESULTS AND DISCUSSION

### Properties of *P. aeruginosa* PA56381 and PA56391 and carbapenemase detection

Two CRPA clinical isolates (PA56381 and PA56391) were consecutively recovered from sputum specimens of two hospitalized patients in a medical ward at a tertiary care hospital in Shanghai, China. PA56381 was isolated on 15 March 2019, followed by PA56391 on 20 March 2019. Both patients had a history of invasive mechanical ventilation. Following invasive mechanical ventilation, both patients received empirical antibiotic therapy with either meropenem or piperacillin-tazobactam. The antibiotic regimen was subsequently shifted to a combination of a cephalosporin and amikacin upon the availability of the initial antimicrobial susceptibility testing report for CRPA (Fig. S1). The clinical isolate *P. aeruginosa* PA56381 exhibited low-level resistance to carbapenems with meropenem and imipenem MIC 8 µg/mL and to most β-lactams. Ceftazidime showed intermediate resistance, but the isolate was susceptible to ciprofloxacin, polymyxin B, amikacin, and ceftazidime-avibactam, with ticarcillin-clavulanate being resistant (Table 1). The modified Hodge test was positive for meropenem and ertapenem but negative for imipenem, suggesting the potential presence of a carbapenemase. Notably, the *bla*_CAE-1_-carrying PAO1 transformant tested positive in the CLSI-recommended mCIM assay, yielding an inhibition zone diameter of 12 mm (within the positive range of 6–15 mm), whereas the empty vector control tested negative (22 mm) and the positive control *K. pneumoniae* ATCC BAA-1705 functioned as expected (Fig. S2).

**TABLE 1:** *In vitro* antimicrobial susceptibility for clinical isolates and recombinant strains producing CAE-1 and KPC-2

Antibiotic	MICs (mg/L)							
	*P. aeruginosa* (PA56381)	*P. aeruginosa* (PA56391)	*E. coli* DH5α (pUCP24)	*E. coli* DH5α (pUCP24-*bla*_CAE-1_)	*E. coli* DH5α (pUCP24-*bla*_KPC-2_)	*P. aeruginosa* PAO1 (pUCP24)	*P. aeruginosa* PAO1 (pUCP24-*bla*_CAE-1_)	*P. aeruginosa* PAO1 (pUCP24-*bla*_KPC-2_)
Piperacillin	64	64	<4	128	256	<4	256	512
Piperacillin-tazobactam	16/4	16/4	<2/4	4/4	128/4	<2/4	128/4	>256/4
Ticarcillin-clavulanate	256/2	>512/2	<4/2	<4/2	256/2	8/2	>512/2	>512/2
Ceftriaxone	NA^*a*^	NA	<0.25	16	8	NA	NA	NA
Ceftazidime	16	16	<0.125	0.25	4	1	64	64
Ceftazidime-avibactam	1/4	1/4	0.06/4	0.125/4	0.06/4	1/4	1/4	2/4
Cefepime	16	16	<0.06	<0.06	2	1	128	>128
Cefoperazone-sulbactam	16	16	<0.25	2	8	2	128	256
Aztreonam	16	16	<0.25	<0.25	32	2	128	512
Imipenem	8	8	<0.06	0.25	4	1	1	64
Meropenem	8	8	<0.016	0.03	1	0.5	4	>32
Ertapenem	32	32	<0.03	<0.03	1	4	8	>64
Levofloxacin	0.25	0.5	<0.125	<0.125	<0.125	0.25	0.25	0.25
Amikacin	4	4	<1	<1	<1	2	2	2
Polymyxin B	1	1	0.25	0.5	0.5	1	1	1

^*a*^ NA, not applicable.

### Divergent resistance phenotypes conferred by CAE-1 in *P. aeruginosa* and *E. coli*

WGS revealed that the two CRPA isolates carried a class A β-lactamase CAE-1. To examine the functionality of *bla*_CAE-1_, the gene was cloned and transformed into *P. aeruginosa* PAO1 and *E. coli* DH5α. The *P. aeruginosa* PAO1 (pUCP24-*bla*_CAE-1_) strain conferred resistance or reduced susceptibility to all β-lactams tested, including piperacillin-tazobactam, ticarcillin-clavulanate, and cefoperazone-sulbactam, but was susceptible to ceftazidime-avibactam. Compared with *P. aeruginosa* PAO1 (pUCP24), MICs of cefepime, piperacillin, ceftazidime, and aztreonam had a 64- to 128-fold increase, while meropenem showed an eightfold increase. The *P. aeruginosa* PAO1 (pUCP24-*bla*_CAE-1_) demonstrated resistance to all three β-lactamase inhibitor combinations (tazobactam, clavulanate, and sulbactam), while remaining susceptible to ceftazidime-avibactam, which indicates the above three β-lactamase inhibitors exhibit no inhibitory activity against CAE-1, whereas avibactam effectively inhibits this β-lactamase. The observed inhibition profiles provide additional evidence that CAE-1 is a new class A carbapenemase.

Cloning of *bla*_CAE-1_ into *P. aeruginosa* PAO1 conferred only an intermediate MIC to meropenem, with no change in imipenem susceptibility. Both clinical isolates showed resistance to meropenem and imipenem. Genomic analysis of the clinical isolates revealed a frameshift mutation (Gly388fs) in the *oprD* gene compared to that of PAO1. The loss of OprD porin production was confirmed by SDS-PAGE. As OprD loss is a well-established mechanism associated with imipenem resistance in *P. aeruginosa* (22). Therefore, the imipenem resistance in these clinical isolates is primarily attributable to OprD inactivation.

The MICs for *E. coli* and *P. aeruginosa* expressing CAE-1 were quite different. Compared to the *P. aeruginosa* PAO1 (pUCP24-*bla*_CAE-1_), the *E. coli* DH5α (pUCP24-*bla*_CAE-1_) strain exhibited significantly lower MICs for all β-lactams, with MIC elevations observed only for ampicillin, piperacillin, cefazolin, cefuroxime, and ceftriaxone (Table 1). To investigate whether the differential resistance profiles between *E. coli* and *P. aeruginosa* transformants were due to variations in gene expression, RT-qPCR was performed (Table S2). Interestingly, *bla*_CAE-1_ expression was lower in *P. aeruginosa* PAO1 compared with *E. coli* DH5α. This indicates that the higher carbapenem MICs observed in *P. aeruginosa* PAO1 are not attributable to overexpression of the carbapenemase but rather to species-specific expression variation (23). The observed resistance profile may result from the host genetic background, where CAE-1 functions synergistically with *P. aeruginosa*’s intrinsic low outer membrane permeability and active efflux systems to prevent antibiotic accumulation more effectively than in the *E. coli* host (24).

### Enzyme kinetics

Kinetic parameters confirmed that CAE-1 hydrolyzed piperacillin, aztreonam, cephalosporins, and carbapenems (Table 2). CAE-1 demonstrated higher catalytic efficiency (*k*cat/*K*m) against piperacillin, aztreonam, and cefepime than against carbapenems and ceftazidime.

**TABLE 2:** Kinetic parameters of CAE-1 and KPC-2

Antibiotic	CAE-1			KPC-2		
	*K*m (μM)^*a*^	*k*cat (s^−1^)^*a*^	*k*cat/*K*m (μM^−1^s^−1^)	*K*m (μM)^*a*^	*k*cat (s^−1^)^*a*^	*k*cat/*K*m (μM^−1^s^−1^)
Meropenem	23.53 ± 3.74	0.35 ± 0.02	0.02	7.91 ± 1.78	4.90 ± 1.16	0.66
Imipenem	30.52 ± 9.34	0.23 ± 0.05	0.01	63.05 ± 7.39	80.51 ± 10.36	1.28
Ertapenem	24.27 ± 3.82	1.71 ± 0.67	0.07	22.07 ± 6.35	11.85 ± 2.00	0.56
Piperacillin	167.38 ± 3.56	95.29 ± 15.36	0.57	21.26 ± 4.61	13.51 ± 2.39	0.64
Ceftazidime	138.72 ± 6.63	3.56 ± 0.38	0.03	61.15 ± 21.75	0.69 ± 0.19	0.01
Cefepime	104.83 ± 10.08	20.07 ± 1.24	0.19	173.53 ± 18.23	17.35 ± 1.57	0.10
Aztreonam	42.07 ± 7.50	8.14 ± 0.73	0.20	92.13 ± 22.95	57.86 ± 16.69	0.62

^*a*^ *k*cat and *K*m values were calculated as the mean + SD of three independent measurements with three different enzyme purifications.

The substrate affinities (*K*m value) of CAE-1 to the three carbapenems were similar, but the substrate turnover rates (*k*cat value) varied, with ertapenem having the highest (1.71 s^−1^) and imipenem the lowest (0.23 s^−1^), leading to significant differences in catalytic efficiency (*k*cat/*K*m): ertapenem (0.07 μM^−1^s^−1^), meropenem (0.02 μM^−1^s^−1^), and imipenem (0.01 μM^−1^s^−1^). This indicates that CAE-1 exhibits high binding affinity for carbapenems but demonstrates limited hydrolytic activity, thereby failing to confer significant resistance (MIC elevation) *in vivo*. According to the catalytic efficiency, piperacillin was the optimal substrate for CAE-1, with a low substrate affinity (*K*m) of 167.38 μM and the highest turnover rates (*k*cat) of 95.29 s^−1^. The catalytic efficiencies of aztreonam and cefepime were similar, at 0.20 μM^−1^s^−1^ and 0.19 μM^−1^s^−1^, respectively. The difference lay in that cefepime had poor affinity (higher *K*m) but high turnover rates, whereas aztreonam had the opposite characteristics. CAE-1 also hydrolyzed ceftazidime, but with a lower substrate affinity (138.72 μM), resulting in a low hydrolysis efficiency of 0.02 μM^−1^s^−1^.

The enzyme kinetic parameters of KPC-2 were simultaneously measured under the same experimental conditions as a reference. CAE-1 demonstrated lower catalytic efficiency against the three carbapenems compared to KPC-2, while displaying approximately comparable efficiency for the other β-lactams tested. This difference in enzymatic activity accounts for the distinct MICs of *bla*_CAE-1_ and *bla*_KPC-2_ in *P. aeruginosa* PAO1.

### Dissemination of *bla*_CAE-1_ among *P. aeruginosa*

**Fig 1:**
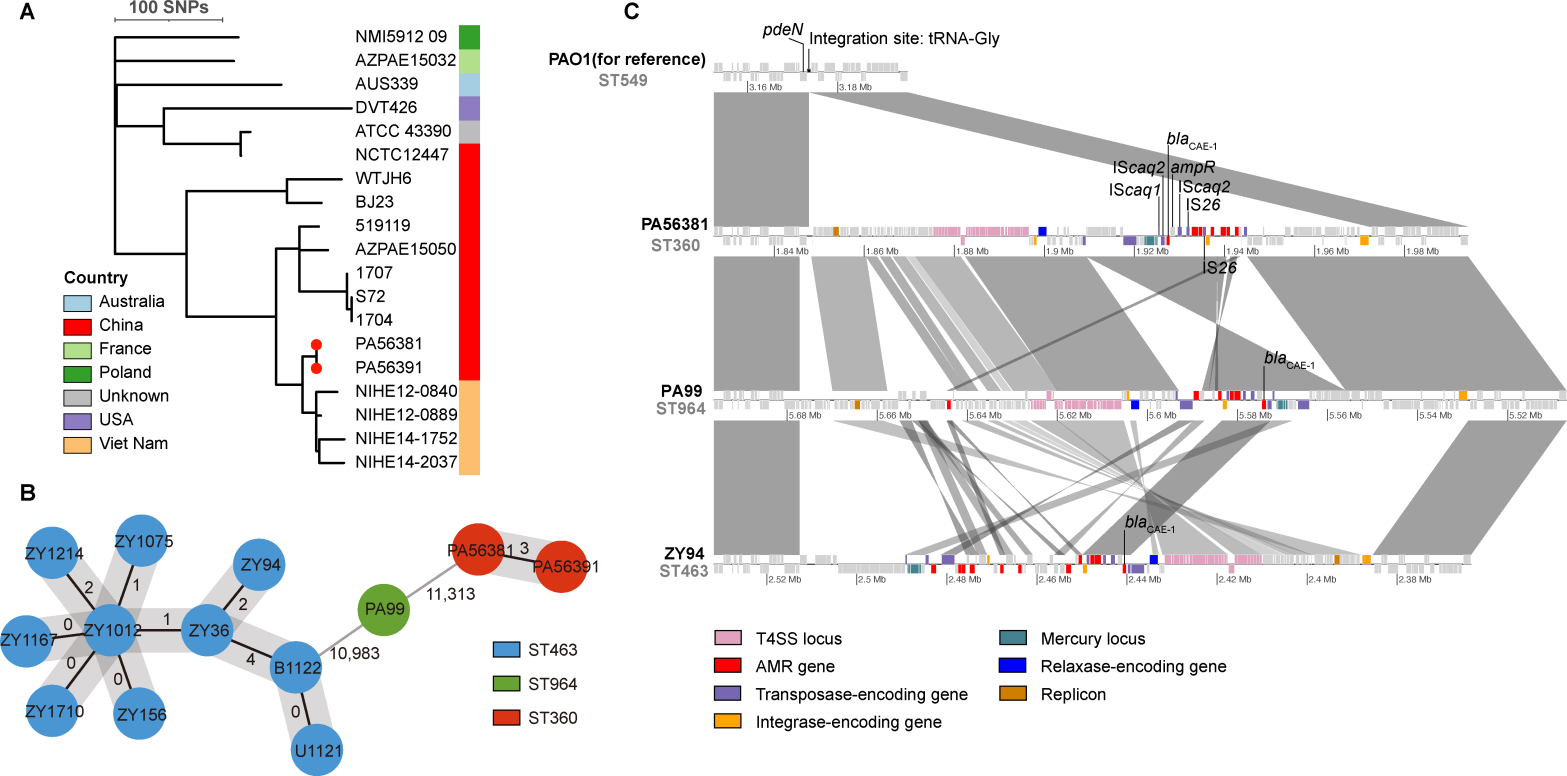
Phylogeny of ST360 isolates and genetic characterization of CAE-1 positive *P. aeruginosa* isolates. (**A**) Phylogeny of 19 ST360 isolates (17 from public database). Two isolates from this study were marked by red dots on the tips. (**B**) Minimum spanning tree based on the cgMLST analysis of 13 CAE-1 positive *P. aeruginosa* isolates. The node colors correspond to the sequence types to which the strains belong. Allele differences were marked adjacent to the edges. (**C**) Comparison of the genetic context of the *bla*_CAE-1_ gene in different *P. aeruginosa* strains (ST360, ST964, and ST463). Strain PAO1 was used as a reference to identify the integration site of the ICE carrying *bla*_CAE-1_. Genes are represented by boxes and colored based on gene function classification. Boxes at the top of the sequence map depict genes on the forward strand, while those at the bottom depict genes on the reverse strand. Gray shadows indicate link regions that share at least 90% sequence identity.

WGS and multilocus sequence typing suggested that the two CRPA isolates carrying *bla*_CAE-1_ belong to ST360. To investigate the evolutionary origins of the two ST360 isolates, we conducted phylogenetic reconstruction using a data set comprising 17 additional ST360 *P. aeruginosa* genomes obtained from the *Pseudomonas aeruginosa* genome database (25). A recombination-filtered core genome phylogeny revealed that PA56381 and PA56391 are closely related (differing by 0 core SNPs), suggesting nosocomial transmission, and form a clade with four Vietnamese isolates collected between 2012 and 2014, with pairwise core SNP distances within the clade ranging from 7 to 23 (Fig. 1A).

A BLASTN search of *bla*_CAE-1_ sequence against *P. aeruginosa* database identified eleven clinical *P. aeruginosa* isolates harboring *bla*_CAE-1_. This cohort included ten ST463 isolates and one ST964 isolate. Subsequent cgMLST analysis revealed that the ten ST463 isolates, along with two ST360 isolates from this study, demonstrated evidence of clonal dissemination (≤4 allele difference) (Fig. 1B).

To gain insight into the mobile genetic elements contributing to the acquisition of *bla*_CAE-1_, the genetic context of *bla*_CAE-1_ in isolates PA56381 (ST360), PA99 (ST964), and ZY94 (ST463) was compared. Notably, *bla*_CAE-1_ was located on an uncharacterized ICE approximately 120 kbp in size, which might result in the potential horizontal transfer of *bla*_CAE-1_ gene among *P. aeruginosa* species (Fig. 1C).

Specifically, *bla*_CAE-1_ is oriented in reverse, positioned immediately upstream of the gene encoding the LysR family transcriptional activator, *ampR*. This chromosomal *bla*_CAE_-*ampR* configuration is structurally analogous to the *ampC-ampR* system observed in Gram-negative bacteria. However, despite this structural similarity, the expression of *bla*_CAE-1_ and *ampR* was not significantly induced by cefoxitin challenge in both clinical isolate PA56381 and *P. aeruginosa* PAO1 transformant harboring *bla*_CAE-1_ and *ampR* (data not shown). Additionally, two *IS*Caq2 elements consistently flank this *bla*_CAE_-*ampR* configuration.

### Inter-species dissemination of *bla*_CAE_

**Fig 2:**
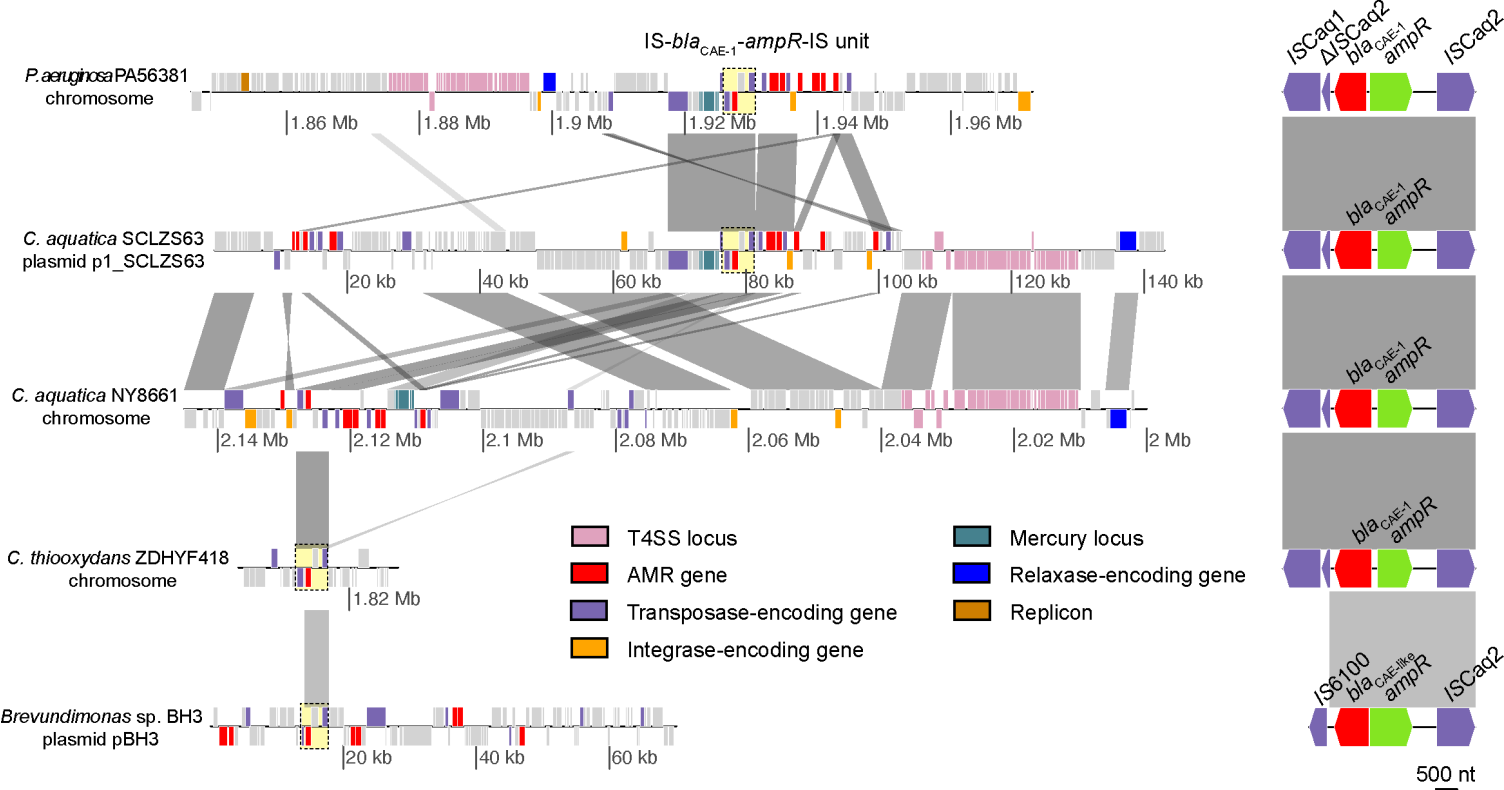
Comparison of the genetic context of the *bla*_CAE_ gene across species. Genes are depicted as boxes (left panel) or arrows (right panel), with colors corresponding to functional classifications. The conserved IS-*bla*_CAE_-*ampR*-IS unit is highlighted by a yellow dashed box in the left panel and further magnified in the right panel.

A BLASTN homology analysis of the *bla*_CAE-1_ against the NCBI GenBank database revealed five additional species beyond *P. aeruginosa* that also carried *bla*_CAE-1_ or *bla*_CAE-like_. The identified genetic elements were distributed across chromosomal locations in *C. aquatica* isolates BB1454 and NY8661 (LR813086.1 and CP096918.1), the plasmid p1_SCLZS63 (CP104280.1) from isolate SCLZS63, and the chromosome of *C. thiooxydans* isolate ZDHYF418 (CP063057.1). In addition, *bla*_CAE-like_ was found on plasmid pBH3a of *Brevundimonas* sp. BH3. The *bla*_CAE-like_ gene on pBH3a exhibited a frameshift mutation compared to *bla*_CAE-1_, resulting from a deletion of an adenine at the 10th nucleotide position, located within the N-terminal signal peptide region. Comparative genomic analysis suggests that the *IS*Caq2 element downstream of the *bla*_CAE_-*ampR* configuration may facilitate the inter-species transfer of *bla*_CAE_ (Fig. 2).

Similar to the well-characterized plasmid-encoded AmpC β-lactamase CMY-2 (26), *bla*_CAE-1_ and its derivatives can be horizontally transferred via plasmids and ICEs. Copy number variation of *bla*_CAE_-harboring plasmids/ICEs may amplify resistance levels, thereby exacerbating clinical threats. Notably, plasmids carrying *bla*_CAE-1_ could also harbor metallo-beta-lactamase gene *bla*_AFM-1_ simultaneously, such as p1_SCLZS63 (27), which might lead to pan-β-lactam resistance, leaving few therapeutic alternatives.

### Conclusion

In conclusion, we identified and characterized the class A carbapenemase CAE-1, which contributed to carbapenem resistance in clinical *P. aeruginosa* isolates. Despite showing moderate hydrolytic efficiency against carbapenems compared to KPC-2, the carbapenemase activity is supported by a positive Hodge test in clinical isolates, a positive mCIM result in a PAO1 transformant, and its characteristic inhibitor profile, which shows resistance to tazobactam, clavulanate, and sulbactam, but strong inhibition by avibactam. The *bla*_CAE-1_-mediated antibiotic resistance differed significantly between *P. aeruginosa* and *E. coli*, indicating species heterogeneity in the expression of CAE-1. CAE-1 has been identified on a conjugative plasmid in *C. aquatica,* which may promote rapid propagation of *bla*_CAE-1_. Potential copy number variation of *bla*_CAE_-harboring plasmids/ICEs might confer high-level β-lactam resistance, thereby exacerbating clinical threats.

## Data Availability

The complete genome sequence of clinical isolate PA56381 and the draft genome sequence of PA56391 have been submitted to the DDBJ/EMBL/GenBank databases under BioProject accession number PRJNA1290494.

## SUPPLEMENTAL MATERIAL

Supplemental material aac.01362-25-s0001.pdf Fig. S1 and S2; Tables S1 and S2.

ASM does not own the copyrights to Supplemental Material that may be linked to, or accessed through, an article. The authors have granted ASM a non-exclusive, world-wide license to publish the Supplemental Material files. Please contact the corresponding author directly for reuse.
