# Supplementary material for: Functional characterization of a novel class A carbapenemase CAE-1 in carbapenem-resistant Pseudomonas aeruginosa clinical isolates
Source: Antimicrob Agents Chemother. 2026 Feb 23;70(4):e01362-25. doi: 10.1128/aac.01362-25 (PMC13041384; doi:10.1128/aac.01362-25)
Supplement: Supplemental material — Fig. S1 and S2; Tables S1 and S2. [file aac.01362-25-s0001.pdf]

Figure S1. Timeline of antibiotic therapy for two patients testing positive for CRPA isolates PA56381 (Patient 1) and PA56391 (Patient 2).

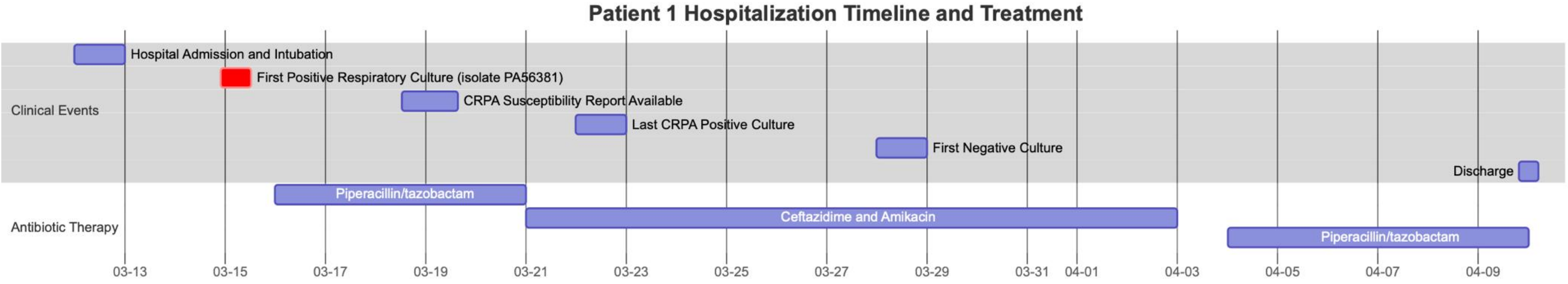

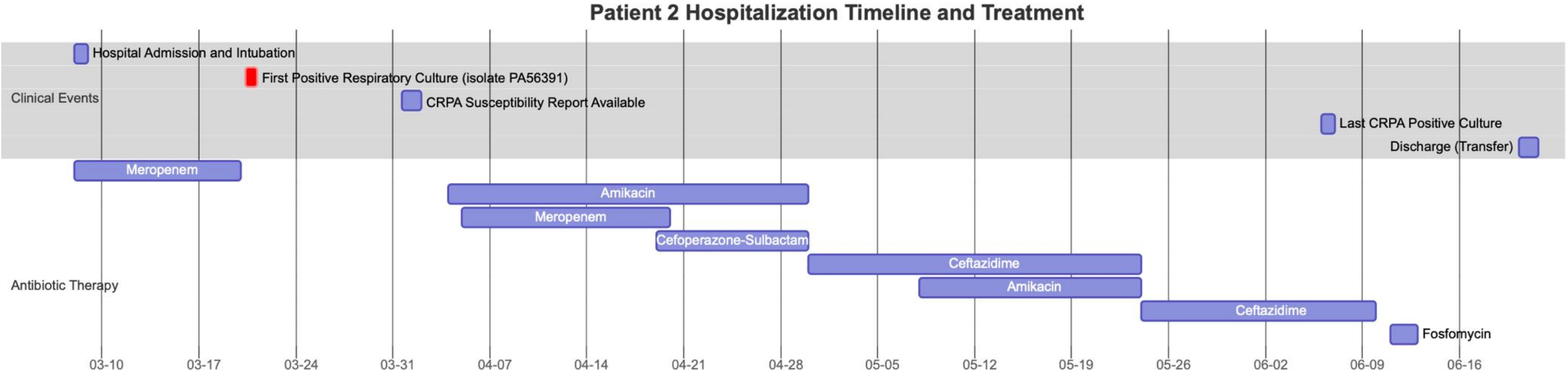

Figure S2.Results of the mCIM test.

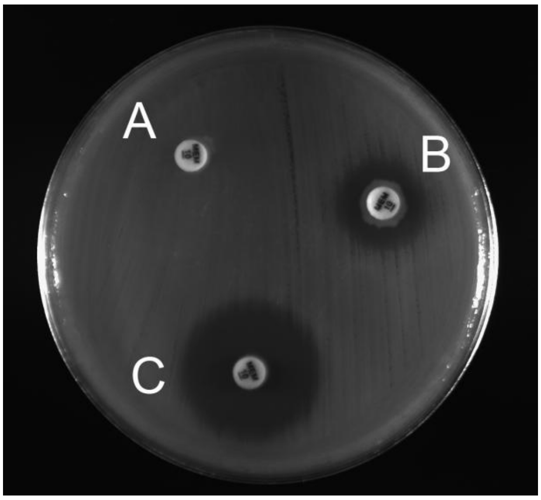

(A) Positive Control (*K. pneumoniae* ATCC BAA-1705, bla_{KPC+}): no zone of inhibition.

(B) Test Strain (*P. aeruginosa* PAO1 pUCP24-bla_{CAE-1}): zone of inhibition is 12mm.

(C) Negative Control (*P. aeruginosa* PAO1 pUCP24): zone of inhibition ≥ 19 mm

Table S1. Bacterial strains, plasmids and primers used in this study.

Strain, plasmid or primer	Relevant characteristics or sequence*	Purpose	Source
Strains			
E. coli			
DH5α	F⁻ φ80*lac*ZΔM15 Δ(*lac*ZYA-*arg*F)U169 *rec*A1 *end*A1 *hsd*R17(rK⁻,mK⁻) *pho*A *sup*E44 λ⁻*thi*-1 *gyr*A96 *rel*A1	Cloning host	Invitrogen
BL21 (DE3)	F⁻ *omp*T *hsd*S_B (r_B^-, m_B^-) *gal dcm* (DE3)	Protein expression host	Invitrogen
P. aeruginosa			
PAO1	A wild-type *P. aeruginosa* strain	Cloning host	A collection of B Holloway[27] and maintained in the laboratory of Dr. Tian Guobao, Sun Yat-sen University
Plasmids			
pUCP24	*Escherichia-Pseudomonas* shuttle vector; gentamicin resistance	Cloning vector	TaKaRa
pUCP24-*bla*$_{CAE-1}$	pUCP24 carrying the full ORF of *bla*$_{CAE-1}$	Antimicrobial susceptibility testing	This study
pUCP24-*bla*$_{KPC-2}$	pUCP24 carrying the full ORF of *bla*$_{KPC-2}$	Antimicrobial susceptibility testing	This study
pET28a (+)	T7 promoter, pBR322 origin; gentamicin resistance	Expression vector	Novagen
pET28a (+)-*bla*$_{CAE-1}$	*bla*$_{CAE-1}$ between NcoI and XhoI sites of pET28a (+)	Overexpression of CAE-1 for purification	This study
pET28a (+)-*bla*$_{KPC-2}$	*bla*$_{KPC-2}$ between EcoRI and PstI sites of pET28a (+)	Overexpression of KPC-2 for purification	This study
Primers			
CAE-1-HindIII-fw	<u>GGCCAGTGCCAAGCT</u>GGGATGTTGGAAGACCCGACC	Cloning of promoter and *bla*$_{CAE-1}$ into plasmids pUCP24	This study
CAE-1-BamHI-rev	<u>CGGTACCCGGGGATC</u>CTATTGCGCCTGATACTGTCGC	Cloning of promoter and *bla*$_{CAE-1}$ into plasmids pUCP24	This study
CAE-1-NcoI-fw	<u>TTTCAGGGCGCCATG</u>AAAACCGAGCGCCACTGG	Cloning of *bla*$_{CAE-1}$ into plasmids pET28a (+)	This study
CAE-1-XhoI-rev	<u>GGTGGTGGTGCTCGA</u>CTATTGCGCCTGATACTGTCGC	Cloning of *bla*$_{CAE-1}$ into plasmids pET28a (+)	This study
Promoter-PstI-fw	<u>CCAAGCTTGCATGCC</u>GGGATGTTGGAAGACCCGAC	Cloning of promoter into plasmids pUCP24	This study
Promoter-rev	<u>AGTGACAT</u>CGTGATCCACCTTTCAAAAAGAAGATGC	Cloning of promoter into plasmids pUCP24	This study

KPC-2-fw	GATCACGATGTCACTGTATCGCCGTCTAGT	Cloning of *bla*$_{KPC-2}$ into plasmids pUCP24	This study
KPC-2-KpnI-rev	ACGAATTCGAGCTCGTTACTGCCCGTTGACGCCCA	Cloning of *bla*$_{KPC-2}$ into plasmids pUCP24	This study
KPC-2-PstI-fw	GCCATGGATCCGGAATTCGCGGAACCATTCGCTAA	Cloning of *bla*$_{KPC-2}$ into plasmids pET28a (+)	This study
KPC-2-EcoRI-rev	GGTGCTCGAGACTGCAGTTACTGCCCGTTGACGCC	Cloning of *bla*$_{KPC-2}$ into plasmids pET28a (+)	This study

[*] Restriction enzyme sites are underline

Table S2: Relative expression levels of *bla*$_{KPC-2}$ and *bla*$_{CAE-1}$ genes.

Species	Reference Gene	Target Gene	Mean Δ Ct[a]	Relative Expression $2^{(-\Delta Ct)}$(Mean ± SD)[b]
E. coli DH5α (pUCP24-*bla*$_{CAE-1}$)	*mdh*	*bla*$_{CAE-1}$	-8.29	319.58 ± 79.05
E. coli DH5α (pUCP24-*bla*$_{KPC-2}$)	*mdh*	*bla*$_{KPC-2}$	-7.71	213.59 ± 52.48
P. aeruginosa PAO1(pUCP24-*bla*$_{CAE-1}$)	*rpsL*	*bla*$_{CAE-1}$	0.9	0.54 ± 0.04
P. aeruginosa PAO1(pUCP24-*bla*$_{KPC-2}$)	*rpsL*	*bla*$_{KPC-2}$	-1.07	2.11 ± 0.09

[a] Mean Δ Ct values represent the average cycle threshold difference normalized to reference genes (*mdh* or *rpsL*) from three biological replicates.

[b] Relative Expression values were calculated as the mean ± SD of three independent biological replicates using the $2^{(-\Delta Ct)}$ method.
